# High‐Performance Strain Sensors Based on Organohydrogel Microsphere Film for Wearable Human–Computer Interfacing

**DOI:** 10.1002/advs.202205632

**Published:** 2022-12-23

**Authors:** Kankan Zhai, Hao Wang, Qiongling Ding, Zixuan Wu, Minghui Ding, Kai Tao, Bo‐Ru Yang, Xi Xie, Chunwei Li, Jin Wu

**Affiliations:** ^1^ Department of Otolaryngology The First Affiliated Hospital of Sun Yat‐Sen University State Key Laboratory of Optoelectronic Materials and Technologies and the Guangdong Province Key Laboratory of Display Material and Technology School of Electronics and Information Technology Sun Yat‐Sen University 510275 Guangzhou P. R. China; ^2^ Department of Rehabilitation Medicine The First Affiliated Hospital Sun Yat‐sen University 510080 Guangzhou P. R. China; ^3^ The Ministry of Education Key Laboratory of Micro and Nano Systems for Aerospace Northwestern Polytechnical University 710072 Xi'an P. R. China

**Keywords:** moisture and frost resistance, organohydrogel microsphere film, ultrasensitive and stretchable strain sensor, wearable human–computer interfacing, wireless sensing system

## Abstract

Stretchable hydrogel‐based strain sensors suffer from limited sensitivity, which urgently requires further breakthroughs for precise and stable human‐computer interaction. Here, an efficient microstructural engineering strategy is proposed to significantly enhance the sensitivity of hydrogel‐based strain sensors by sandwiching an emulsion‐polymerized polyacrylamide organohydrogel microsphere membrane between two Ecoflex films, which are accompanied by crack generation and propagation effects upon stretching. Consequently, the as‐developed strain sensor exhibits ultrahigh sensitivity (gauge factor (GF) of 1275), wide detection range (100% strain), low hysteresis, ultralow detection limit (0.05% strain), good fatigue resistance, and low fabrication cost. In addition, the sensor features good water, dehydration, and frost resistance, enabling real‐time strain monitoring in various complex conditions due to the encapsulation of Ecoflex film and the addition of glycerol and KCl. Through further structural manipulation, the device achieves superior response to tiny strains, with a GF value of 98.3 in the strain range of less than 1.5%. Owing to the high strain sensing performance, the sensor is able to detect various human activities from swallowing to finger bending even under water. On this basis, a wireless sensing system with apnea warning and single‐channel gesture recognition capabilities is successfully demonstrated, demonstrating its great promise as wearable electronics.

## Graphical abstract

An ultrasensitive, stretchable, and environmental‐tolerant strain sensor based on crack mechanism with an Ecoflex‐microspheres‐Ecoflex sandwich structure is created successfully. It features ultra‐high sensitivity (gauge factor: 1275), wide detection range (0–100%), ultralow detection limit, wide operating temperature range, and good waterproofness. A wireless monitoring system is also developed, providing a more effective strategy to realize human–computer interfacing.

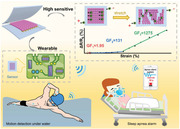

## 1 Introduction

Recently, wearable and portable sensing electronics have attracted a lot of attention and have greatly driven the development of Internet of Things.^[^ ^1^, ^2^, ^3^, ^4^, ^5^, ^6^ ^]^ Among them, flexible strain sensors that can fit on human skin or clothes can adapt to various mechanical deformations and convert them into detectable electrical signals in real time, and can be widely used in motion monitoring, gesture recognition, physiological signal monitoring, etc., playing an important role in human–computer interaction.^[^ ^7^, ^8^, ^9^, ^10^, ^11^, ^12^, ^13^, ^14^ ^]^ For more efficient detection, the most important thing is that the strain sensor needs to simultaneously meet the requirements of high sensitivity and wide strain detection range.^[^ ^15^ ^]^ Wherein, the sensitivity of the strain sensor can be assessed by the gauge factor (GF), which is defined as: GF = (*R−R*_0_)/*R*_0_/*ε*, where *R*, *R*_0_, and *ε* represent the instantaneous resistance, the initial resistance, and the applied strain, respectively, representing the relative resistance variation under unit strain.^[^ ^16^ ^]^ The sensitivity of a strain sensor depends largely on its sensing mechanism. In general, the generation and propagation of cracks in rigid conductive films or the disconnection between constituent elements in the network structure can lead to a sharp change in resistance, ultimately resulting in ultrahigh sensitivity in the sensing material.^[^ ^17^, ^18^ ^]^ In addition, the wide strain detection range of the sensor is highly desirable not only for the monitoring of large human motions (≈50%) but also for subtle human motions (<1%), which can greatly broaden the application versatility of the device. Thus, the flexibility and stretchability of the strain sensor need to be improved to enhance the mechanical compliance and broaden the strain detection range. To address these, intensive endeavor has been devoted to developing stretchable strain sensors by integrating or embedding a variety of highly conductive nanomaterials, including metal nanoparticles, Ag nanowires, MXene, carbon nanotubes (CNTs), graphene, onto elastic polymer substrates, exhibiting high‐performance strain sensing capability.^[^ ^19^, ^20^, ^21^, ^22^, ^23^, ^24^ ^]^

Despite these advances, there are some inherent challenges in the development of strain sensors. First, high sensitivity and wide strain sensing range are a dilemma in current strain sensors. For crack‐based strain sensors, the ultra‐high sensitivity originates from the crack generation mechanism.^[^ ^7^ ^]^ With further crack propagation, the material will be irreversibly damaged, generally with a limited strain detection range (<5%).^[^ ^25^, ^26^ ^]^ For example, Wang et al. demonstrated an ultrasensitive crack‐based strain sensor based on a gold/titanium (Au/Ti) thin film, with a GF value of up to 5000 in the range of 0–1% strain.^[^ ^26^ ^]^ With regard to the 2D nanomaterial‐based strain sensors, including Ag nanowires, CNTs, graphene nanosheets, etc., their stretchability can even be improved to 100%, mainly originating from the disconnection and dispersion between these nano‐building blocks.^[^ ^19^, ^27^, ^28^ ^]^ Unfortunately, their sensitivity is significantly reduced, and the conductive network still remains highly interconnected in the large stretched state, thus resulting in a smaller resistance change. Secondly, the fabrication process and cost of these traditional sensing materials are relatively high, and they are prone to irreversible damage during stretching due to their non‐stretchability and exhibit poor mechanical compliance. Novel cost‐efficient and intrinsically stretchable sensing materials need to be developed while ensuring accurate and stable strain sensing. Finally, underwater strain detection is in urgent demand, which is of great practical significance especially in swimming sports, and the environmental tolerance of the device needs to be improved to broaden the scope of application scenarios. On the whole, it is challenging to develop low‐cost and reliable wearable strain sensors with high sensitivity and full detection range that can work in various environments.

Recently, hydrogel‐based strain sensors have been widely reported because of its softness, stretchability, transparency and good biocompatibility, enabling repetitive and reversible responses to extremely large strains.^[^ ^29^, ^30^, ^31^, ^32^, ^33^ ^]^ For example, Zhang et al. reported a strain sensor based on NaCl/sodium alginate (SA)/polyacrylamide (PAM) supramolecular hydrogels, showing an extremely broad strain detection range.^[^ ^34^ ^]^ However, the strain responsiveness of this hydrogel is inferior, and the GF value only reaches 2.7 in the strain range of 200–1800%. The sensing mechanism can be attributed to the elongated ion transmission pathway in the stretched hydrogel, with less resistance change. Besides, these strain sensors always have unsatisfactory limit of detection (LOD) and cannot be exploited for tiny strain detection. In various human activities, the sensitive detection of subtle deformations including pulse, heartbeat, swallowing and vocalization, often shows greater practical significance and can be widely used for health monitoring, disease diagnosis, and human–computer interaction. To address these, nanocomposite hydrogels have been extensively constructed by dispersing various nanofillers such as CNTs, graphene, and MXene in ionic conductive hydrogels to form a highly interconnected electronic conductive network.^[^ ^3^, ^35^, ^36^, ^37^, ^38^ ^]^ Upon stretching, the sensor sensitivity is significantly improved, normally with GF over 10, resulting from the tunneling effect and disconnection between electronic conductive building blocks. Nonetheless, the cost of these devices increases accordingly, and the hydrogel has poor anti‐freeze and anti‐drying properties and will swell in water, leading to its failure in various harsh environments such as polar, desert, or underwater.^[^ ^39^, ^40^, ^41^, ^42^, ^43^ ^]^ Thus, further breakthroughs in the sensitivity of pure hydrogel‐based strain sensors are urgently needed for more cost‐effective and accurate motion monitoring.

In this work, inspired by conventional crack‐based strain sensors, we developed a resistive‐type stretchable strain sensor by utilizing PAM organohydrogel microsphere film as the novel sensing layer for the first time, which exhibits ultra‐high sensitivity (with the highest GF of 1275 among existing hydrogel‐based sensors), ascribed to the disconnection between microspheres and the generation and propagation of cracks in the sensing layer upon stretching. Wherein, the diameters of the microspheres separated from the soak solution are distributed between 50 and 700 µm, and they are connected by weak reversible hydrogen bonds due to the residual trace of solvent. Therefore, the sensing layer can still return to the initial state after suffering a large crack, resulting in a relatively wide strain detection range. In addition to these, the sensor also exhibits good fatigue resistance, low hysteresis, good frost resistance and thermal stability. Also, the sensitivity of the sensor exhibits a width dependence, as the width of the sensing layer is reduced from 8 to 2 mm, the sensitivity of the sensor to tiny strains increases to 98.3 owing to the easier generation of cracks. It is found that the content of KCl and glycerol (Gly) in the organohydrogel can greatly affect the sensitivity of the sensor. Only when the KCl concentration and the volume ratio of glycerol to water reached 0.1 m and 1:1, respectively, the sensor exhibits relatively low initial resistance (*R*_0_) and large resistance change (Δ*R*) during stretching, thus leading to the optimal response. On account of the excellent strain sensing performance and waterproof elastomeric encapsulation layers, the sensor performed accurate, stable and real‐time motion monitoring even in water. As a demonstration, a wireless sensing system based on the developed organohydrogel microsphere crack‐based strain sensor and a specific circuit is developed, enabling real‐time apnea alarm and single‐channel gesture recognition. Overall, this study provides a novel and efficient idea to fabricate high‐performance hydrogel‐based sensors, which can play an important role in health monitoring, soft robotics, and artificial intelligence.

## 2 Results and Discussion

### 2.1 Characterizations of PAM Organohydrogel Microspheres and Device

**Figure 1:**
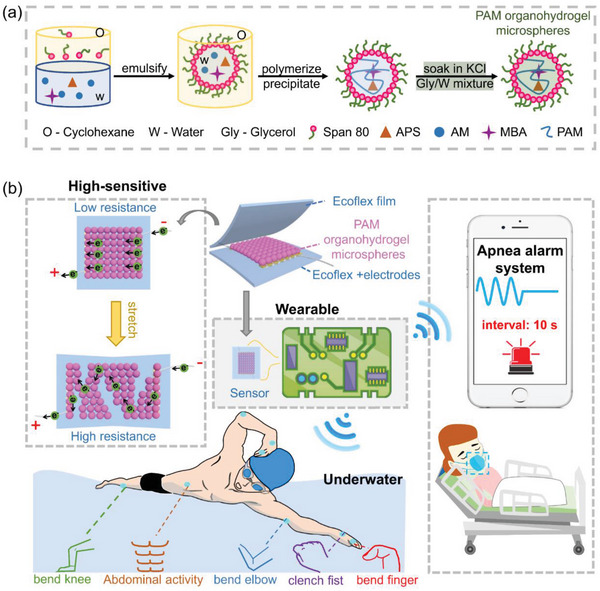
Fabrication processes and application prospect of the PAM organohydrogel microsphere membrane‐based strain sensor. a) Schematic illustration of the fabrication process of the PAM organohydrogel microspheres. b) Scheme illustrating the structure and working mechanism of the sandwich‐structured strain sensor, and it can be used as a wireless wearable device for real‐time monitoring of various human activities even in water as well as apnea alarm.

In this study, numerous PAM organohydrogel microspheres were prepared by utilizing a simple and efficient inverse emulsion polymerization, with the details described in the experiment part. In this strategy, as illustrated in **Figure** **1**a, the aqueous phase containing AM monomer, APS initiator and MBA crosslinker, and the oil phase comprising amphiphilic Span 80 were prepared separately. Once they were mixed and vigorously stirred, the amphiphilic Span 80 molecules self‐assembled in the system, in which the hydrophilic ends aggregated inward to interact with water molecules, while the hydrophobic ends were exposed at the periphery to form interfaces with cyclohexane molecules. Eventually, a large number of water emulsion droplets were formed and uniformly distributed in the continuous oil phase, existing as a water‐in‐oil emulsion. After in situ polymerization, a large number of PAM hydrogel microspheres can be collected by filtering them from the unpolymerized oil phase. Finally, PAM organohydrogel microsphere membrane containing a binary solvent system was formed by soaking the hydrogel microspheres in KCl/Gly/water mixtures, and some soaking solution remained between the microspheres, resulting in a better connection between the microspheres due to the formation of hydrogen bonds between hydroxyl groups of glycerol and amide groups of PAM chains, as shown in Figure S1, Supporting Information. During tensile process, the reversible weak hydrogen bonds are broken following the separation of microspheres. After the strain is released, these hydrogen bonds are able to reform with the approach of microspheres, providing a good stability to the PAM microsphere membrane. By sandwiching this microsphere membrane between two Ecoflex films, highly sensitive crack‐based strain sensor was constructed, and it could be used not only for real‐time motion monitoring even in water but also for sleep apnea monitoring when combined with IoT technology (Figure 1b), which will be discussed in detail later. Respectively, the microsphere membrane as a sensing layer induces cracks and leads to a sharp change in resistance after stretching, while the elastic Ecoflex films enable the reconnection of the crack edges after strain release, ensuring the stretchability and self‐recovery of the device. It is worth noting that the Ecoflex films should be treated by air plasma at 180 W for 300 s to improve the hydrophilicity of the surfaces and prevent electrodes from shedding. As shown in Figure S2, Supporting Information, the water contact angle of Ecoflex film changes from 106° to 35.3° after the hydrophilic treatment, indicating that the hydrophilicity of the film has been greatly improved.

**Figure 2:**
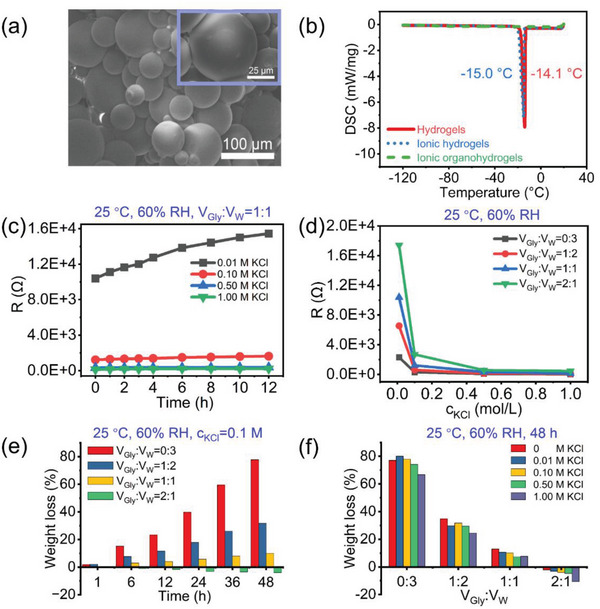
a) SEM images of the dried microspheres. b) DSC curves of the hydrogels, ionic hydrogels, and ionic organohydrogel microspheres. c) Resistance evolution of a series of organohydrogels immersed with defined volume ratios of Gly/water (1:1) and different KCl concentrations (0.01, 0.1, 0.5, and 1 m) when exposed to 25 °C and 60% RH for different time. d) Resistance curves of the organohydrogels with different volume ratios of Gly/water (0:3, 1:2, 1:1, and 2:1) versus the soaked KCl concentration from 0.01 to 1 m. e) Water‐retaining ability of the PAM organohydrogels with 0.1 m KCl concentration and different volume ratios of Gly/water under 25 °C and 60% RH. f) Weight loss of the organohydrogels with different KCl concentration (0, 0.01, 0.1, 0.5, and 1 m) and different volume ratios of Gly/water (0:3, 1:2, 1:1, and 2:1) after placing at 25 °C for 48 h.

**Figure** **2**a shows the scanning electron microscope (SEM) image of the dried PAM organohydrogel microspheres. It can be seen that the diameters of individual microspheres range from 10 to 100 µm and appear as smooth surfaces. Generally, hydrogels have poor environmental tolerance due to the presence of a large amount of free water, and they can easily freeze and dry at sub‐zero temperatures or after prolonged storage, which is not conducive to its application in various environments.^[^ ^44^ ^]^ Excellently, the introduction of Gly can greatly enhance the frost and drying immunity of hydrogels through the displacement of free water and the formation of hydrogen bonds with surrounding water molecules.^[^ ^45^, ^46^ ^]^ Here, the differential scanning calorimetry (DSC) spectra of the PAM hydrogel, the ionic hydrogel with KCl addition, and the ionic organohydrogel with KCl and Gly are compared in Figure 2b, and it can be found that the freezing point of the organohydrogel is the lowest, dropping below −120 °C. While the ionic hydrogel shows a similar freezing point compared to pristine hydrogel, indicating the crucial role of Gly in improving the freezing resistance of the hydrogel.

Pure hydrogels have poor conductivity due to the limited number of ions,^[^ ^47^ ^]^ and a good ionic conductivity is generally required for hydrogel‐based strain sensors. Here, both KCl and Gly additions have a huge impact on the material conductivity. As shown in Figure 2c and Figure S3a–c, Supporting Information, when the volume ratio of Gly/water in the immersed mixture is fixed, the conductivity of the organohydrogel microsphere membrane can be significantly promoted by increasing the KCl concentration in the soaking solution from 0.01 to 1 m. However, the conductivity decreases with the storage time due to the evaporation of water, despite that this phenomenon can be alleviated by increasing the concentration of KCl. The addition of insulating Gly can also effectively inhibit the dehydration of hydrogel, but has a detrimental effect on the conductivity of the system. Figure 2d comprehensively demonstrates the resistance of a series of organohydrogel microsphere after immersion in mixtures containing different KCl concentrations (0.01, 0.1, 0.5, and 1 m) and volume ratios of Gly/water (0:3, 1:2, 1:1, and 2:1). The results show that when the KCl concentration is the same, the resistance of the resulting microsphere membrane increases with increasing Gly proportion, originating from the lower ionic mobility in Gly.

Synergistically, both KCl and Gly play a positive role in enhancing the moisturizing properties of the organohydrogel microspheres. To explore these, a series of samples soaked in a mixture containing 0.1 m KCl and different volume ratios of Gly/water (0:3, 1:2, 1:1, and 2:1) were placed in an environment of 25 °C and 60% RH, and their masses were recorded at intervals (Figure 2e). Without the introduction of Gly, the mass loss of the obtained ionic hydrogel reached 75% after standing for 48 h, reflecting the poor stability. After the introduction of Gly, the mass loss was greatly slowed down, which can be attributed to the consequent reduction of the vapor pressure in the system and the fact that water molecules are bound in the network by forming hydrogen bonds with Gly molecules.^[^ ^48^, ^49^ ^]^ Especially when the volume ratio of Gly/water was 2:1, the material was even able to absorb surrounding water molecules during placement, as the vapor pressure of the system is reduced below that of surrounding. Likewise, increasing the ionic content can also alleviate water loss to some extent due to the ionic hydration effect.^[^ ^50^ ^]^ Figure 2f shows the comparison of the total weight loss of each organohydrogel microspheres with different KCl concentrations and specific Gly/water volume ratio after staying at 25 °C, 60% RH for 48 h. Clearly, the mass loss decreased with increasing KCl concentration, indicating enhanced water retention capabilities. As a result, with the synergistic effect of KCl and Gly, the obtained PAM organohydrogel microspheres show satisfactory environmental tolerance and conductivity simultaneously, enabling long‐term and stable application in strain sensing in various environments.

### 2.2 Characterizations of Strain Sensing Performance

**Figure 3:**
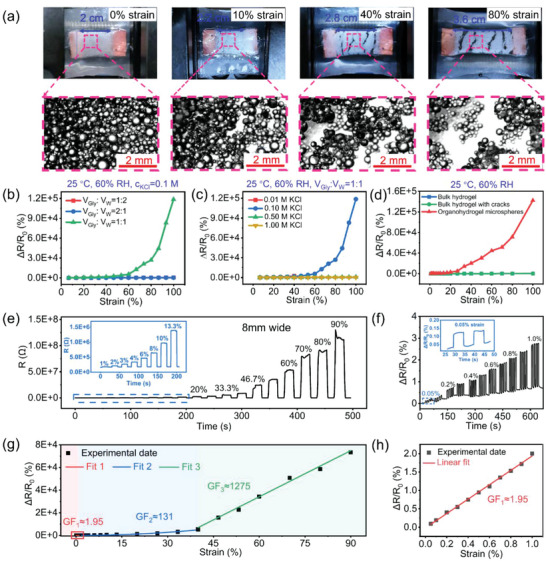
Sensing mechanism and electromechanical response of the crack‐based strain sensor. a) Photographs (above) and optical microscope images (below) illustrating crack evolution of the organohydrogel microsphere membrane at 0%, 10%, 40%, and 80% strains, respectively. b) Typical resistance response versus strain curves of the sensors with different volume ratios of Gly/water (1:2, 1:1, and 2:1). c) Typical response versus strain curves of the sensors with different KCl concentrations (0.01, 0.1, 0.5, and 1 m). d) Comparison of response versus strain curves of strain sensors based on bulk hydrogel and organohydrogel microsphere membrane. e) Dynamic resistance curve of the strain sensor with 0.1 m KCl and 1:1 volume ratios of Gly/water to different strains (1–90%), the inset is an enlarged view of the dynamic resistance curve of the sensor to 1–13.3% strains. f) Dynamic response of the sensor to different tiny strains (0.05–0.1%). g) The response–strain curve of the sensor and its linear fitting curves in different strain regions, the GF values are calculated as 1.95, 131, and 1275 in the strain ranges of 0.05–1%, 1–40%, and 40–90%, respectively. h) The enlarged view of the response‐strain curve and corresponding linear fitting curve of the sensor to 0.05–1% strains.

Here, utilizing the PAM organohydrogel microsphere membrane as the sensing layer, a crack‐based strain sensor was constructed by encapsulating it between two Ecoflex films, and the electrodes were connected at both ends. As shown in **Figure** **3**a, the size of microspheres was distributed between 50 and 700 µm after being soaked in Gly/water solution, and the appearance of the sensing layer changes when subjected to different tensile strains. With increasing strain, multiple cracks gradually generated, propagated, and merged with each other to eventually form penetrating cracks, leading to the increase in the resistance and finally the disconnection of the circuit. More details can be observed through their optical microscope images. When there is no applied strain, the PAM organohydrogel microspheres are tightly assembled without cracks, and the internal ions can easily pass through the whole membrane. Upon stretching to 10% strain, part of the microspheres is separated from each other under the action of external force, and some tiny cracks perpendicular to the stretching direction are generated. There are a large number of microsphere islands that exist in the gap and are closely connected to each other to maintain the transport of ions. With further stretching, the strain is mainly concentrated in the interstitial region, and the crack gradually widens. At the beginning, the microsphere islands in the gap are separated from each other, and the microsphere bridges are developed between the islands, enabling the poor transport of ions. Later, the microsphere bridges will elongate and fracture with increasing strain due to slippage between the microspheres. When the strain reaches 80%, the whole membrane is divided into several parts by forming several wide penetrating cracks, with only a few microsphere bridges remaining in the gap, and the ion transport is seriously hindered. Consequently, the crack propagation mechanism of the sensing layer leads to rapid changes in the overall resistance, enabling ultra‐sensitive strain detection.

Considering the significant effect of Gly and KCl on the conductivity of the organohydrogel microsphere membrane, the strain responsiveness can also be severely affected by these additives. To investigate this, first, the dynamic resistance response curves of a series of strain sensors based on the PAM organohydrogel microsphere membranes with different volume ratios of Gly/water (1:2, 1:1, and 2:1) were obtained (Figure S4a–c, Supporting Information), where the KCl concentration remained the same. It can be found that the resistance of the device increases rapidly after being subjected to tensile strain due to the reduction of the conduction channel, which is consistent with the strain‐dependent morphological change of the sensing layer. After the strain is released, the resistance of the sensor can be restored to the initial state due to the re‐contact of the crack edge during the spring back of the Ecoflex film. Due to the increase of insulating Gly, the initial resistance of the sensor gradually increases from 25 to 90 kΩ as the volume ratio of Gly/water increases from 1:2 to 2:1 (Figure S5, Supporting Information). By comparing their response–strain curves (Figure 3b), we found that the strain response is greatest when the volume ratio of Gly/water is 1:1, superior to the other three by three orders of magnitude. Subsequently, by fixing the volume ratio of Gly/water is 1:1, strain sensors with different KCl concentrations (0.01, 0.1, 0.5, and 1 m) were also compared, and their dynamic resistance response curves to different strains and corresponding response–strain curves were shown in Figure 3c and Figure S4d–f, Supporting Information. As can be seen, when the KCl concentration and the volume ratio of Gly/water are fixed at 0.1 m and 1:1, respectively, the response of the strain sensor is optimal, reaching as high as 118 300% for 100% strain. Fundamentally, this significant influence of composition on the responsiveness can be mainly attributed to its modulation of ionic conduction in the material.

During the conduction process, extensive ion transport channel and excellent transport efficiency are essential for a good conductivity. Wherein, the transport channel mainly reflects the connection density between the internal units of the material, depending on the microstructure of the material, while the ion migration efficiency is closely related to the solvent properties and ionic numbers in the material. In this case, the strain responsiveness is based on microsphere rearrangement and crack generation and propagation, that is, there is a gradual reduction of the ion transport channel in the organohydrogel microsphere membrane during stretching, resulting in an increase in its resistance. In order to achieve improved response, the initial resistance should be reduced (Figures S4 and S5, Supporting Information). Considering these, when the Gly content is relatively low, ions can easily cross between microspheres due to better contact and higher ionic mobility, with low initial resistance, which is conducive to higher responsiveness. Whereas when the Gly content is too high, it is highly non‐conductive due to the insulating properties of Gly. And there is a large amount of Gly remaining between adjacent microspheres due to its high viscosity, making these microspheres difficult to completely separate with each other and form cracks during stretching (Figure S6c, Supporting Information), thus resulting in an unsatisfactory response. Similarly, an increase in KCl would also favor an improved response due to a lower initial resistance. Whereas, although the transport efficiency remains essentially constant during stretching, it will determine the modulation strength of the final resistance by the transport channel. For materials with high ion transport efficiency, even if partial cracks are generated, the remaining ionic transport channels are sufficient for the device to have a good conductivity, which is why the response decreases at low Gly content and high KCl concentration. As shown in Figure S7a,b, Supporting Information, we compared the responses of the bulk PAM hydrogels with and without cracks to different strains, with continuous ion transport channel and high transport efficiency due to the structural continuity. The results show that their strain responsiveness is similar but small, indicating the necessity of moderate ion transport efficiency for high responsiveness. In particular, the response of the strain sensor based on the microsphere membrane is much higher than that of continuous bulk hydrogel (Figure 3d), as well as state‐of‐the‐art hydrogel‐based strain sensors, demonstrating the superiority of the crack propagation mechanism compared to traditional geometric change mechanism in improving the strain sensitivity of hydrogels.

Figure 3e shows the dynamic resistance response curve of the strain sensor to different strains from 1% to 90%, demonstrating its promising potential for full‐range strain sensing detection, as the tensile strain of human skin is less than 100%. In particular, it can respond accurately and repeatedly to even small strains of less than 1% (Figure 3f), showing an extremely low LOD (0.05% strain), which is rare in hydrogel‐based strain sensors. From the corresponding response–strain curves and their linear fitting curves (Figure 3g,h), it can be seen that the GF values of the strain sensor in the strain ranges of 0.05–1%, 1–40%, and 40–90% are estimated to be 1.95, 131, and 1275, respectively. This difference comes from the different arrangement of the microspheres in the sensing layer to adapt to different tensile deformations. As for the microsphere membrane we developed, it is not a single‐layer ordered microsphere arrangement, but involves the close packing of multiple layers of microspheres. When the strain sensor begins to suffer from minimal strain (<1%), closely packed microspheres will slip with each other and spread out in the stretching direction, resulting in a reduction in overlapping area. In this case, the change in resistance is mainly related to the rearrangement of the packed microspheres without the creation of cracks, resulting in a small strain sensitivity. With gradually increased strain, the GF value showed a great improvement due to the gradual generation and propagation of fine cracks and the rapid reduction of conductive channels, enabling a drastic change in resistance. As the applied strain continues to increase, the crack propagates to traverse the entire sensing layer, and a slight tensile growth at this stage will cause the separation of the remaining microsphere bridge in the gap regions, resulting in an ultra‐high sensitivity.

**Figure 4:**
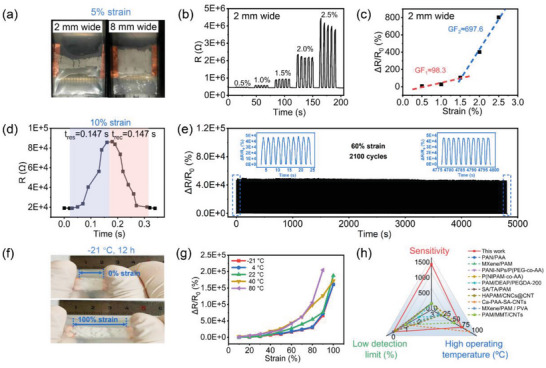
a) Photographs of sensors with 2 and 8 mm wide sensing layers at 5% strain. b) Dynamic response of the 2 mm wide strain sensor to small strains of 0.5–2.5%. c) The response–strain curve of the 2 mm wide strain sensor and its linear fitting curve in different strain ranges. d) Analyses of the response (*t*_res_) and recovery time (*t*_rec_) of the strain sensor from the dynamic response–recovery curve to 10% strain. e) The durability test of the strain sensor under repeated strain of 60% for 2100 cycles. f) Photographs of the microsphere membrane‐based sensor before and after being stretched to 100% after placing at −21 °C for 12 h. g) Response versus strain curve of the sensor at different temperatures. h) Performance comparison of the developed sensor with other typical hydrogel‐based strain sensors, including the sensitivity, LOD, and operating temperature range.

Note that the response of the sensor to extremely small strains can be greatly improved by reducing the width of the sensing layer from 8 to 2 mm. As shown in **Figure** **4**a, after being subjected to a 5% strain, the 2 mm wide sensing layer already developed some obvious penetrating cracks, while the 8 mm wide sensing layer remained largely intact. In this case, there are not enough microspheres in the narrow sensing layer to slip with each other in the stretching direction to accommodate the deformation of the device, thereby causing premature cracking during stretching. As a result, sensors with narrow sensing layers can achieve ultra‐high responses to tiny strains, but the strain detection range of the device is greatly compromised. Figure 4b,c shows the dynamic response curve of the strain sensor with a 2 mm wide sensing layer in response to different repeated strains and the resulting response–strain curves. As expected, the GF values of this sensor are greatly improved to 98.3 in the strain range of 0.5% to 1.5%, and 697.6 in the strain range of 1.5% to 2.5%, suggesting great application potential in the perception of subtle human motions. Therefore, based on this width‐dependent sensitivity characteristic, we can modulate the strain sensitivity and detection range by adjusting the width of sensing layer, which can adapt to different application scenarios.

Excellently, this strain sensor exhibits an extremely fast response and recovery speeds and a good fatigue resistance, which are all indispensable conditions for evaluating whether it can be applied in practice. When subjected to 10% strain, both the response and recovery time were determined to be 0.147 s (Figure 4d), which was basically consistent with the tensile speed. This means that our sensor can respond to strain instantly without hysteresis, and thus it can be used for real‐time human–machine interaction after being combined with a specific circuit system. To demonstrate the durability of the sensor, a cyclic test with 60% strain for up to 2100 cycles was carried out, as shown in Figure 4e. The invariable response to strain throughout the cyclic test demonstrated an excellent fatigue resistance, repeatability and the capability for long‐term and stable strain monitoring. Further, continuously repeated performance tests of a sensor for six days and the performance tests of a series of sensors prepared at the same condition were also performed, as shown in Figures S8 and S9, Supporting Information. The same initial resistances (about 40 kΩ) and small errors of response to strain for different sensors and the same sensor at different time all demonstrated the good controllability and stability of our sensors. In addition to these, the developed strain sensor exhibits a wide operating temperature range due to the excellent frost resistance and thermal stability of the PAM organohydrogel microspheres, capable of being applied in various harsh conditions. After placing the sensor at −21 °C for 12 h, the sensor still maintains good tensile properties, and some obvious cracks are still formed on the sensing layer under 100% strain (Figure 4f), which enables a normal response based on the crack generation and propagation. To investigate the strain sensing performance of the sensor at different temperatures, the sensors were first placed at −21, 4, 22, 40, and 80 °C for 1 h before testing, and then their dynamic response curves to strains from 10% to 100% were recorded, as shown in Figure S10, Supporting Information. It can be seen that they all display superior strain responsiveness at different temperatures, but the maximum strain drops to 90% at 80 °C. As shown in Figure 4g, their response–strain curves at different temperatures were compared, and the GF values in the 50–80% strain range were calculated as 664, 647, 1235, 2331, and 2719 at −21, 4, 22, 40, and 80 °C, respectively. Obviously, the sensitivity of the sensor at high temperature is higher than that at low temperature. At low temperature, the initial resistance of the sensor is not only increased due to the reduced ion mobility (Figure S11, Supporting Information), but also the formation of cracks is slightly suppressed due to the enhanced intermolecular forces between the microspheres, both leading to a reduction in strain sensitivity. At elevated temperature, the sensor also has an increased initial resistance, as the water in the microspheres will escape and condense on the internal surface of the Ecoflex film. The difference is that the hydrogen bonds between the microspheres will be broken at high temperature, so that the microspheres in the sensing layer are more likely to separate from each other, thus resulting in an increase in strain sensitivity as well as a decrease in the maximum detection strain.

Here, we quantitatively compare the various performance parameters of our developed sensor with that of previously reported hydrogel‐based strain sensors, including the sensitivity, LOD, and operation temperature range, as shown in Figure 4h and Table S1, Supporting Information. Notably, the strain sensitivity of our sensor is excellent, two or three orders of magnitude better than other sensors. In addition, our sensor also features a wide strain detection range and an extremely low LOD. Especially, its response sensitivity in the low strain range (<1.5%) can reach 98.3, achieving a breakthrough in the detection of tiny strain. Even compared with carbon‐based strain sensors, the strain sensitivity of our sensor is still comparable, and even has a wider strain detection range than theirs. Satisfactorily, this excellent strain sensing performance makes the developed strain sensor an excellent candidate for efficient motion monitoring under various scenarios in practice.

### 2.3 Wearable Applications of Strain Sensors

**Figure 5:**
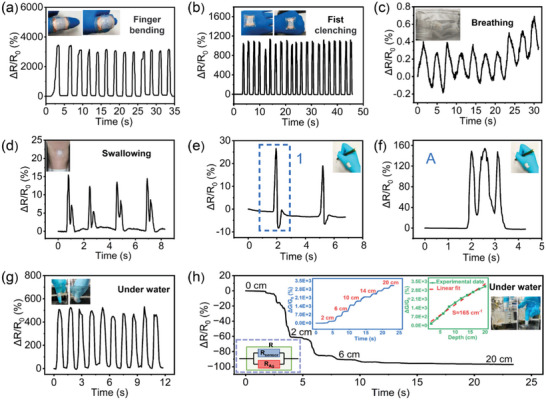
Demonstration of various applications of the organohydrogel microsphere sensor. Dynamic response of the sensor to a) finger bending, b) hand clenching, c) breathing, and d) swallowing. Dynamic response of the sensor attached to the hand when writing different symbols: e) “1” and f) “A.” g) Dynamic response of the sensor to finger bending in water. h) Dynamic resistance response of the sensor under different depth of water, the insets are the dynamic conductance response curve and corresponding response versus water depth curve, and the equivalent circuit model.

Since our organohydrogel microsphere membrane‐based strain sensor is highly sensitive, soft and stretchable, it can be utilized as wearable electronics in human–computer interaction by attaching it to different parts of the human body with double‐sided tape. As shown in **Figure** **5**a, the sensor is fixed on the thumb, and the bending of the joint is sensitively and repeatedly detected, showing an ultra‐high response of 3000. Similarly, hand clenching can be detected by attaching the sensor to the back of the hand, and its response is also high up to around 1000 (Figure 5b), showing a good application potential in real‐time monitoring of tiny motion. Further, based on the effect of the expiratory airflow on the sensor, the sensor attached to the mask can output regular signals during exhalation and inhalation (Figure 5c), providing a simple and effective method for respiratory monitoring, which can play an important role in the diagnosis and treatment of some diseases. As for subtle human motions like swallowing, it can also be precisely monitored with our sensors by attaching it to the throat (Figure 5d). In particular, when different letters and numbers are written, the sensor attached to the hand exhibits different characteristic response peaks (Figure 5e,f; Figure S12, Supporting Information), providing a method for simple character recognition without the need for sophisticated instruments and complex algorithms. In the following research, the sensor can be combined with the robotic arm to achieve a high degree of human–machine interaction, which has broad promising application possibilities in industry, service industry, and healthcare.

Underwater motion monitoring is an urgent need and plays a vital role in the motion and healthy monitoring of swimmer, underwater robot sensing systems, and underwater disturbance detection.^[^ ^51^, ^52^ ^]^ Stretchable strain sensors can be exploited to transduce underwater mechanical behaviors including gestures to electrical signals conveniently.^[^ ^53^, ^54^ ^]^ Unfortunately, pristine hydrogel‐based strain sensors are difficult to apply in underwater as it will swell in water. Here, due to the encapsulation of the hydrophobic Ecoflex film, the developed strain sensor exhibits excellent water resistance. As shown in Figure 5g, the sensor attached to the finger can still repeatedly respond to finger bending under water. Although the response drops to around 450 due to the pressure of the surrounding water, which alleviates the crack formation to a certain extent, it is still superior to other reported hydrogel‐based strain sensors. Concomitantly, the device can also measure the water depth. As the sensor‐fixed finger gradually increases its depth in water from 0 to 20 cm, the dynamic resistance and converted conductance response curves of the sensor are shown in Figure 5h. It can be found that with the increase of water depth, the conductance of the sensor gradually increases with a corresponding decrease in resistance. From conductance response versus water depth curve, there is a positive proportional relationship between them, the sensitivity calculated from the slope of the curve is estimated to be 165. One possible explanation is that at different water depths, the water pressure makes the contact between the microspheres in the sensing layer tighter, resulting in a smaller resistance. However, small changes of microsphere membrane under the hydraulic pressure are not enough to provide such a high level of responsiveness, so there must be other reasons for the remarkable change in resistance.

To explore this, the exposed Ag wires were replaced with the rubber‐covered Ag wires to prepare the strain sensor. As the sensor gradually increases its depth in water from 0 to 14 cm, the dynamic resistance and converted conductance response curves and conductance response versus water depth curve of the sensor are shown in Figure S13, Supporting Information. The highest sensitivity calculated from the curve is estimated to be 3.8, which is much lower than the sensor with exposed Ag wires, demonstrating the important role of exposed Ag wires in improving the sensitivity of the sensor under water. Through further consideration, we conjecture that the change in resistance is also related to the change in the conductive path. As illustrated in the insets of Figure 5h, when the exposed Ag wire is immersed in water, a new conductive path is formed between the Ag wires in water, connected in parallel with the sensor. The resistance of the newly formed conductive path increases with water depth due to the larger cross‐sectional area, enabling sensitive water depth monitoring. This is not the case for the rubber‐covered Ag wires since they cannot form additional conductive path in water. As a consequence, it is anticipated that sensors attached to various parts of the human body can be used for the real‐time monitoring of motion and water depth during swimming considering their excellent strain responsiveness and water depth detection capabilities, so that human health and safety can be guaranteed.

**Figure 6:**
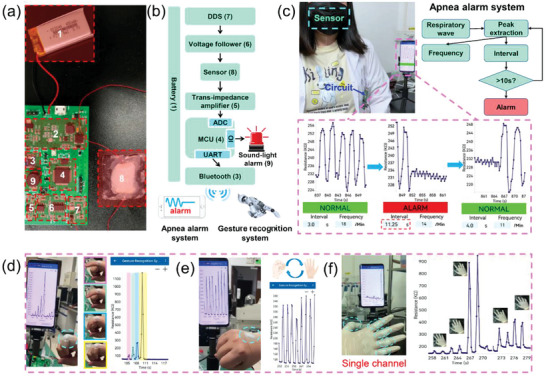
Wireless human–computer interaction system based on the strain sensor. a) Photograph of the wireless sensing system, including a sensor, rechargeable lithium battery, and specifically designed PCB. b) Schematic illustrating the structure and function of the wireless sensing device. c) The wireless respiration monitoring and apnea alarm: the signal “Normal” (green) showed in the phone program when breathing is normal, and turned to “Alarm” (red) when the breath stops, along with a buzzer. In addition to the dynamic response curves, the interval and frequency of breath are also displayed on the phone. The wireless single‐channel gesture recognition system to recognize d) the bending of fingers at different angles, e) the extending and clenching of the hand, and f) the bending of different fingers by connecting five individual sensors in series and attaching them to five fingers.

As a proof of concept, a specific wireless human–computer interaction system was designed to implement apnea alarm and gesture recognition. As shown in **Figure** **6**a, the developed sensor is connected to a designed printed circuit board (PCB) with signal conversion, processing, and wireless transmission functionalities, and the system is powered by a rechargeable lithium battery. Figure 6b carefully illustrates the composition and working principle of each module of the sensing system. In detail, a 200 Hz, 600 mV sine wave signal can be generated by the DDS module and subsequently boosted by a voltage follower, which is then applied across the strain sensor. When subjected to strain, the current signal output by the sensor is amplified and converted into a voltage signal by the transimpedance amplifier. Finally, the processed signal is collected by the Analog Digital Converter (ADC) of STM32 and sent to the terminal device via Bluetooth module. With the integration of the sensor on the mask, the sensing system can realize real‐time respiratory monitoring, and the waveform corresponding to the breathing process can finally be displayed in the self‐programming application of the Smartphone (Figure 6c; Movie S1, Supporting Information). Further, the respiratory rate can be obtained and displayed by programming a specific algorithm on the terminal device. When apnea is detected for more than 10 s, the system will alarm immediately, providing an effective means to realize early diagnosis and auxiliary treatment of patients with sleep apnea syndrome. By fixing the sensor on the finger or the back of the hand, signals corresponding to the different bending states and stretching of the fingers as well as the opening and clenching of hands can be displayed and recognized in real time on the terminal device (Figure 6d,e; Movies S2 and S3, Supporting Information). Furthermore, five sensors with different sensitivities were obtained by changing the width of the sensing layer, and then they were connected in series and attached to five fingers respectively to achieve simple gesture recognition with a single channel (Figure S14, Supporting Information). This is advantageous to traditional gesture recognition using five channels for signal acquisition and processing due to its simple and cost‐effective attributes. As shown in Figure 6f and Movie S4, Supporting Information, the bending of different fingers corresponds to different response peaks, which are derived from the different bending conditions of these fingers and the different sensitivities of the sensors attached to them. Therefore, we can identify the excited sensor based on the magnitude of the resistance change across the circuit, and thus the bent finger.

## 3 Conclusion

In summary, PAM organohydrogel microspheres with diameters ranging from 50 to 700 µm were prepared by emulsion polymerization followed by immersion in a KCl/Gly/water mixture. By sandwiching a stable conductive membrane assembled from these microspheres between two Ecoflex films, a crack‐based strain sensor capable of reversibly and stably responding to tensile deformation was constructed, enabling ultra‐high responsiveness to both large and tiny strains through the modulation of the sensing layer width, with GF values of 1275 and 98.3 in the strain ranges of 40–90% and 0–1.5%, respectively. This represents state‐of‐the‐art performance standards of hydrogel‐based sensors. By introducing KCl and Gly, the conductivity, anti‐drying and anti‐freezing ability of the obtained organohydrogels were greatly improved, ensuring the excellent environmental tolerance of the strain sensor. The effects of KCl and glycerol on the sensitivity of the final sensor were also explored, and the optimal KCl concentration and volume ratio of Gly/water were determined to be 0.1 m and 1:1, respectively. The superior sensitivity can be attributed to the disconnection between the microspheres and the crack propagation in the sensing layer during stretching. As a result, the strain sensor can perform sensitive detection of a variety of human activities such as joint movements, swallowing and breathing even underwater. Thanks to the encapsulation of the Ecoflex films, the sensor can operate normally in water, capable of sensitive underwater motion monitoring and water depth detection. Moreover, we also developed a wireless sensing system for apnea alarm and gesture recognition by combining specific circuits with the wearable sensors, providing a more effective method to realize human‐machine interaction and diverse healthcare applications.

## 4 Experimental Section

### Synthesis of PAM Organohydrogel Microspheres

The PAM organohydrogel microspheres were prepared through an efficient inverse emulsion polymerization method, and all chemicals used here have not undergone further purification. The monomer (AM, 10 wt%), initiator (APS, 1 wt%), cross‐linking agent (MBA, 1 wt%) were dissolved in 50 mL of DI water as water phase, and the emulsifier span 80 (4 wt%) was dissolved in 50 mL of cyclohexane as oil phase. A stable emulsion was formed under high speed (1500 rpm s^−1^) stirring. Then, the emulsion was placed in a 250 mL of three‐mouth bottle with the water bath. The temperature of water bath was set to 60 °C, and the whole reaction process was under the condition of nitrogen atmosphere and condensate water for 3 h. After in situ polymerization, the hydrogel microspheres were precipitated in the anhydrous ethanol several times to remove the residual cyclohexane on the surface of the microspheres. Finally, the PAM organohydrogel microspheres were obtained by soaking the dried hydrogel microspheres in mixtures containing different concentrations of KCl and different volume ratios of Gly/water for a period of time.

### Fabrication of Strain Sensors

The strain sensor exhibited a sandwich structure consisting of two Ecoflex films and a sensing microsphere layer in the middle. First, two Ecoflex films were obtained by spin‐coating the prepolymerized mixture and then curing at room temperature, and their thickness could be effectively controlled by changing the spin coating speed and time. Before assembly, the surface of the Ecoflex film was treated with air plasma cleaner to render it hydrophilic at 180 W for 300 s, and electrodes were pre‐placed on both sides. Then, a temporary rectangular slot mold with dimensions of 20×8×2 mm was assembled on this Ecoflex film with several 2 mm thick rectangular glass sheets, and the quantified PAM organohydrogel microspheres were uniformly filled therein. Subsequently, the loose layer of microspheres was pressed to the height of the mold using a glass sheet, whereby the uniform PAM organohydrogel microsphere film with a fixed size and thickness was formed after removing the glass sheets. Finally, another Ecoflex film was covered on the obtained sensing layer, and the edge was fixed on the first Ecoflex film with prepolymerized mixture to obtain a stable strain sensor. Additionally, the thickness of the sensing layer and the density of microspheres therein could be controlled by varying the thickness of the assembled mold and the mass of the filled microspheres.

### Material Characterizations

The morphology and size of organohydrogel microspheres and strain sensors were characterized by a scanning electron microscope (XL 30 ESEM, Philips) and an optical microscope (MOTIC, Shangguang, Beijing); Relative resistance variation was measured by an LCR digital bridge (TH2382 LCR Meter, Tonghui, Changzhou). The tensile testing was carried out on a precision electric tensile test bench (WDM‐500, Weidu, Zhejiang) at a deformation rate of 5 mm s^−1^ at 25 °C; The DSC spectra were tested on a differential scanning calorimeter (Netzsch, DSC‐204 F1) at a cooling rate of 5 °C min^−1^ from 20 to −120 °C with nitrogen flow.

### Study Participation

Prior to participation in the experiments, informed consent was obtained from the volunteer in all experiments.

## Conflict of Interest

The authors declare no conflict of interest.

## Supporting information

Supporting Information

Supplemental Movie 1

Supplemental Movie 2

Supplemental Movie 3

Supplemental Movie 4

## Data Availability

The data that support the findings of this study are available from the corresponding author upon reasonable request.
