# Supplementary material for: High‐Performance Strain Sensors Based on Organohydrogel Microsphere Film for Wearable Human–Computer Interfacing
Source: Adv Sci (Weinh). 2022 Dec 23;10(6):2205632. doi: 10.1002/advs.202205632 (PMC9951583; doi:10.1002/advs.202205632)
Supplement: Supplementary file 1 — Supporting Information [file ADVS-10-2205632-s004.pdf]

Supporting Information

High-performance strain sensors based on organohydrogel microsphere film for wearable human-computer interfacing

Kankan Zhai,[#] Hao Wang,[#] Qiongling Ding,[#] Zixuan Wu, Minghui Ding, Kai Tao, Bo-Ru Yang, Xi Xie, Chunwei Li,* Jin Wu**

K. Zhai, H. Wang, Q. Ding, Z. Wu, B. Yang, X. Xie, C. Li, J. Wu
Department of Otolaryngology, The First Affiliated Hospital, State Key Laboratory of Optoelectronic Materials and Technologies and the Guangdong Province Key Laboratory of Display Material and Technology, School of Electronics and Information Technology
Sun Yat-Sen University
Guangzhou, 510275, PR China
E-mail: wujin8@mail.sysu.edu.cn; hi_chunwei@aliyun.com
K. Tao
The Ministry of Education Key Laboratory of Micro and Nano Systems for Aerospace
Northwestern Polytechnical University
Xi'an, 710072, PR China
E-mail: taokai@nwpu.edu.cn
M. Ding
Department of Rehabilitation Medicine, The First Affiliated Hospital
Sun Yat-sen University
Guangzhou, 510080, PR China

[#] These authors contributed equally to this work.

This section includes:

- **Figure S1.** Schematic illustrating the hydrogen bonds formed between hydroxyl groups of glycerol and amide groups of PAM chains may increase the adhesive strength between PAM microspheres.
- **Figure S2.** Water contact angles of Ecoflex film before and after the hydrophilic treatment with plasma.
- **Figure S3.** Resistance variation of PAM organohydrogel microspheres soaked in mixtures with different KCl concentrations (0.01, 0.1, 0.5, and 1 M) as a function of storage time under 25 °C and 60% RH.
- **Figure S4.** Dynamic resistance response curves of strain sensors based on the PAM organohydrogel microsphere with different volume ratios of Gly/water and KCl concentrations.
- **Figure S5.** Initial resistance bar graph of the sensors with different volume ratios of Gly/water (1:2, 1:1, and 2:1).
- **Figure S6.** Optical microscope images and photos of the organohydrogel microsphere membrane with different volume ratios of Gly/water.
- **Figure S7.** Dynamic response curves of strain sensors based on bulk hydrogel without and with cracks.
- **Figure S8.** The resistance response of the four sensors to different strains.
- **Figure S9.** The resistance response of the sensor to different strains for six days.
- **Figure S10.** Dynamic response of sensors to 10%-100% strains at different temperatures.
- **Figure S11.** The effect of temperature on the initial resistance of the strain sensor.

- **Figure S12.** Dynamic response of the sensor attached to the hand when writing different symbols.
- **Figure S13.** The resistance and conductance response of the sensor with rubber-covered wires to different depth of water.
- **Figure S14.** The circuit diagram of the single-channel gesture recognition system for recognizing the bending of different fingers.
- **Table S1.** The performance index of various hydrogel-based strain sensors corresponding to the radar diagram in **Fig. 4h**.

Other Supplementary Material for this manuscript includes the following:

- **Movie S1.** Video demonstrates the wireless respiration monitoring and apnea alarm with the strain sensor.
- **Movie S2.** Video depicts the recognition of the bending of fingers at different angles using the wireless single-channel gesture recognition system.
- **Movie S3.** Video shows the recognition of the extending and clenching of the hand using the wireless single-channel gesture recognition system.
- **Movie S4.** Video demonstrates the monitoring of the movement of five fingers respectively using the wireless single-channel gesture recognition system.

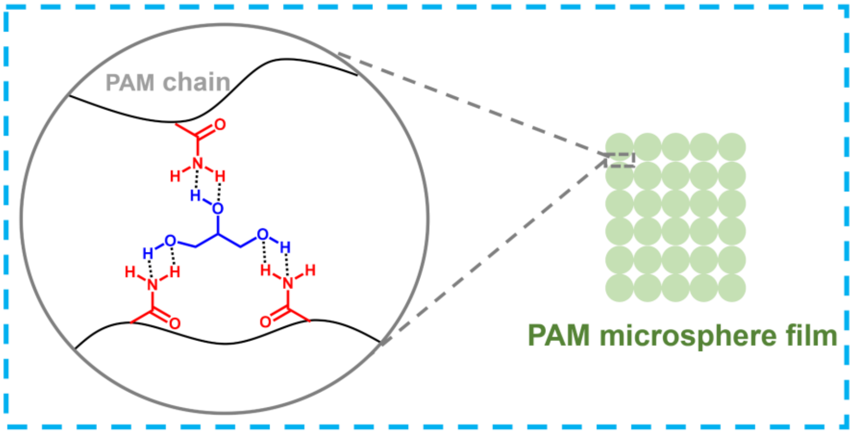

Figure S1. Schematic illustrating the hydrogen bonds formed between hydroxyl groups of glycerol and amide groups of PAM chains may increase the adhesive strength between PAM microspheres.

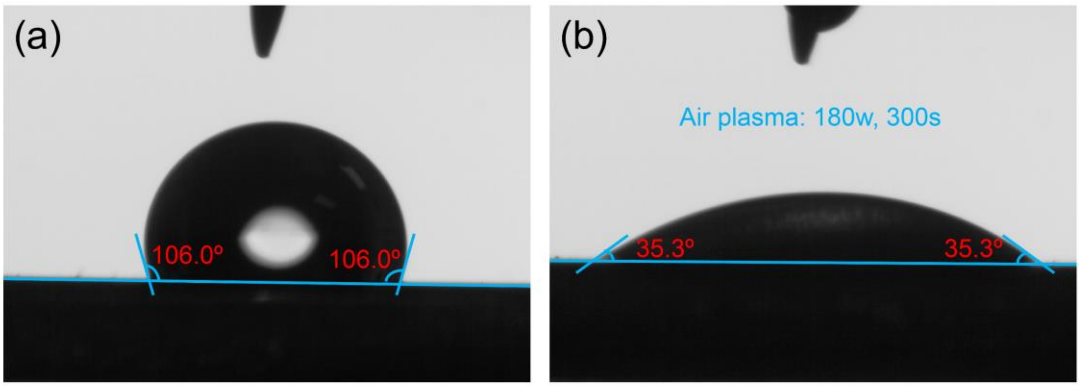

Figure S2. Water contact angles of Ecoflex film (a) before and (b) after the hydrophilic treatment with plasma.

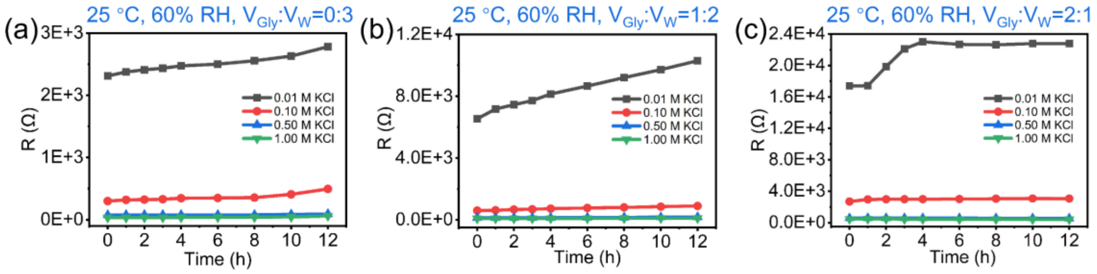

Figure S3. Resistance variation of a series of PAM organohydrogel microspheres soaked in mixtures with a fixed volume ratios of Gly/water (a) 0:3, (b) 1:2, (c) 2:1 and different KCl concentrations (0.01, 0.1, 0.5, and 1 M) as a function of storage time under 25 °C and 60% RH.

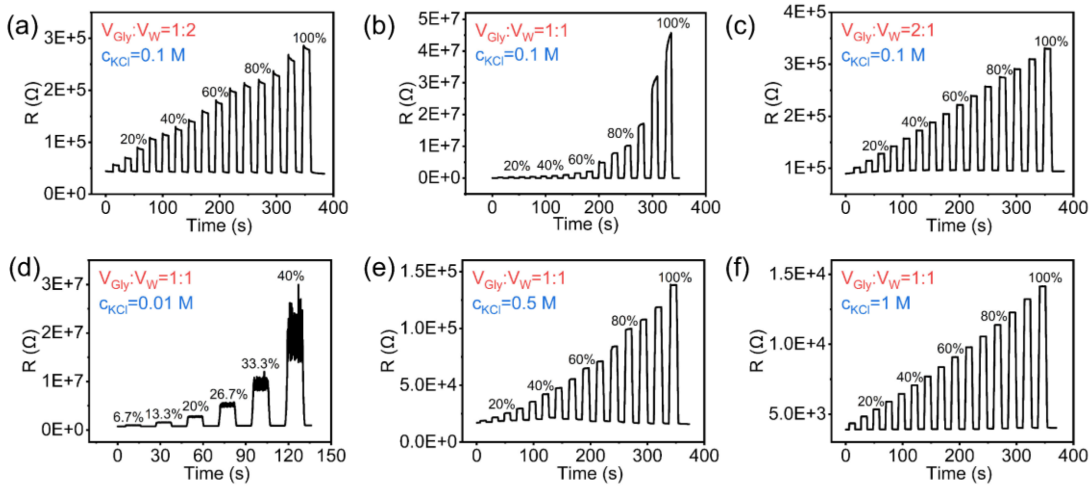

Figure S4. Dynamic resistance response curves of a series of strain sensors based on the PAM organohydrogel microsphere membranes with (a-c) different volume ratios of Gly/water (1:2, 1:1, and 2:1) and (d-f) different KCl concentrations (0.01, 0.1, 0.5, and 1 M).

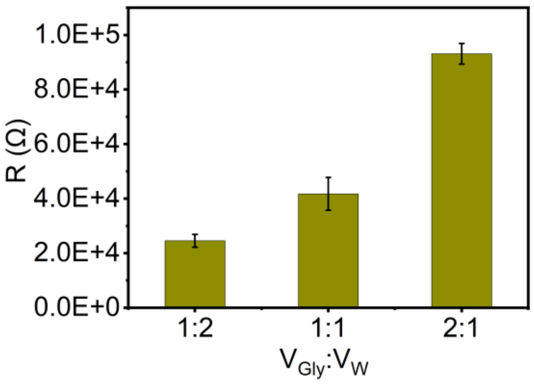

Figure S5. Initial resistance bar graph of the sensors with different volume ratios of Gly/water (1:2, 1:1, and 2:1).

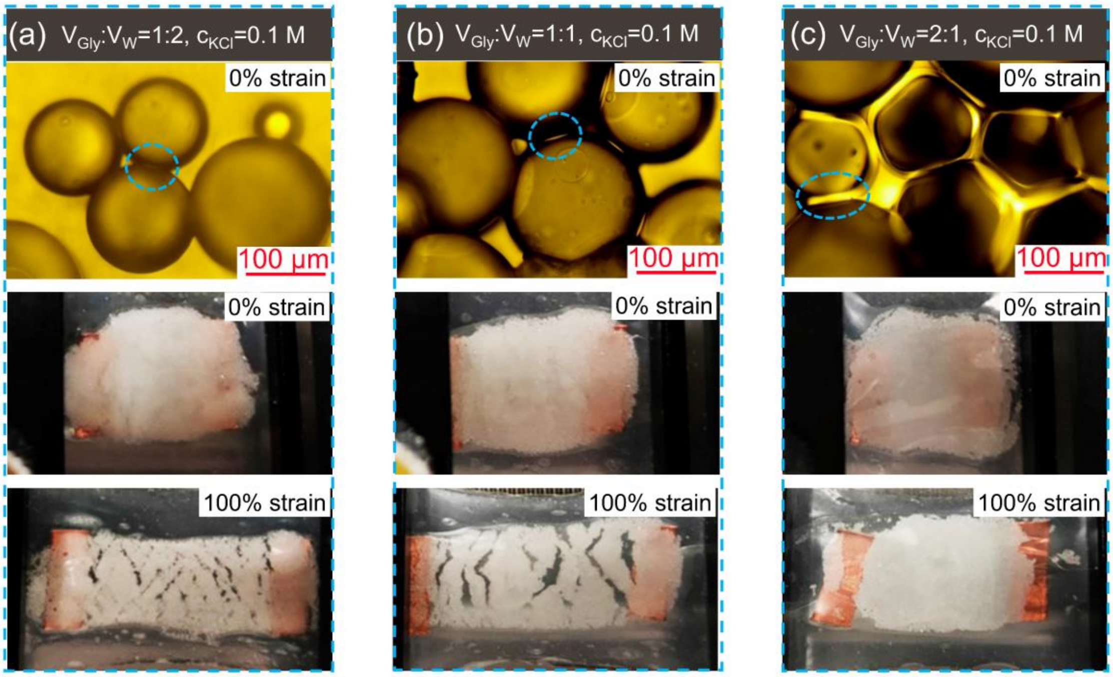

Figure S6. Optical microscope images (above) and photographs (below) of the organohydrogel microsphere membrane with a fixed KCl concentration (0.1 M) and different volume ratios of Gly/water: (a) 1:2, (b) 1:1 and (c) 2:1.

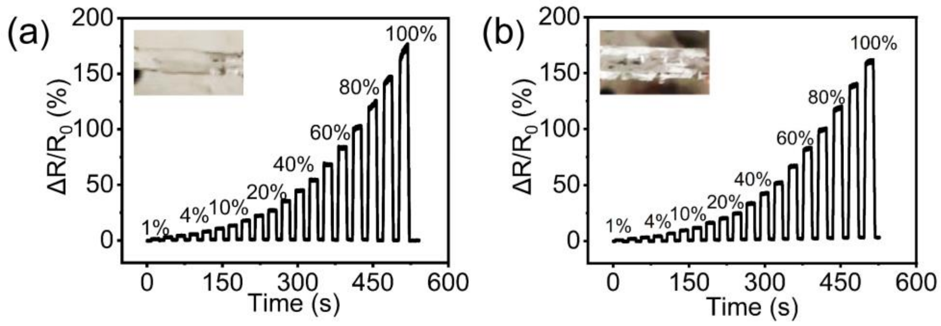

Figure S7. Dynamic response curves of strain sensors based on bulk hydrogel (a) without cracks and (b) with cracks.

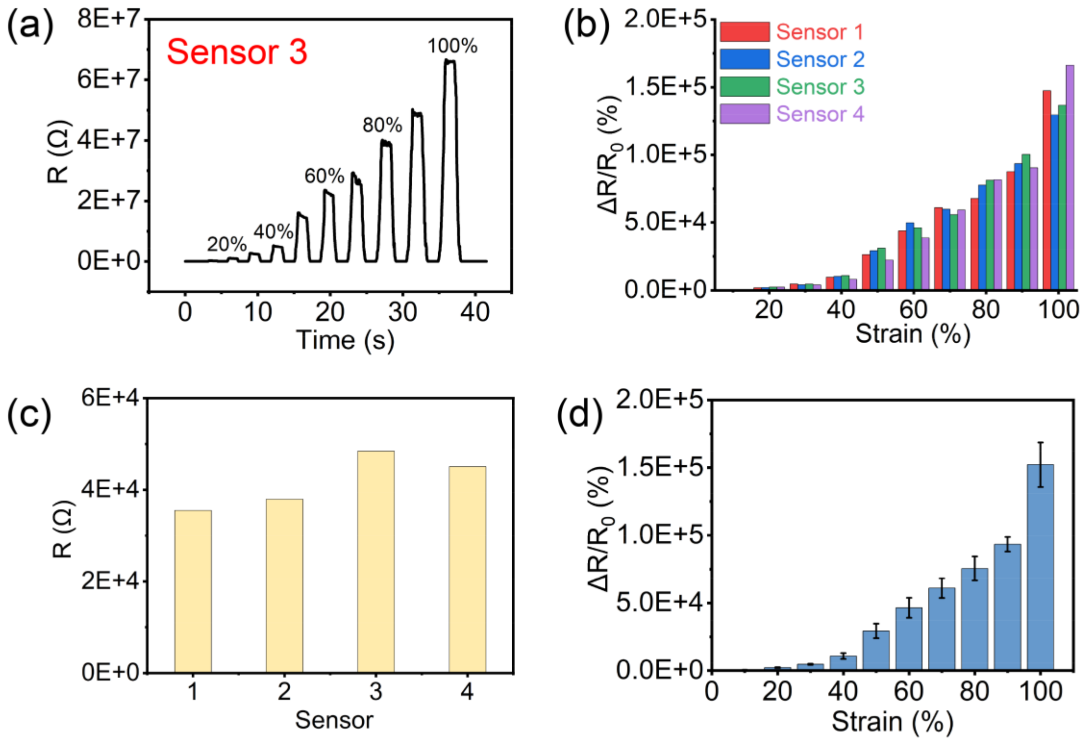

Figure S8. (a) Dynamic resistance variation curve of the sensor 3 in response to different strains (1-100%). (b) Resistance response versus strain bar graphs of sensor 1, sensor 2, sensor 3 and sensor 4, which were fabricated in different batch with the same experimental procedures. (c) The initial resistance of four sensors. (d) The average resistance response of the four sensors to various strains.

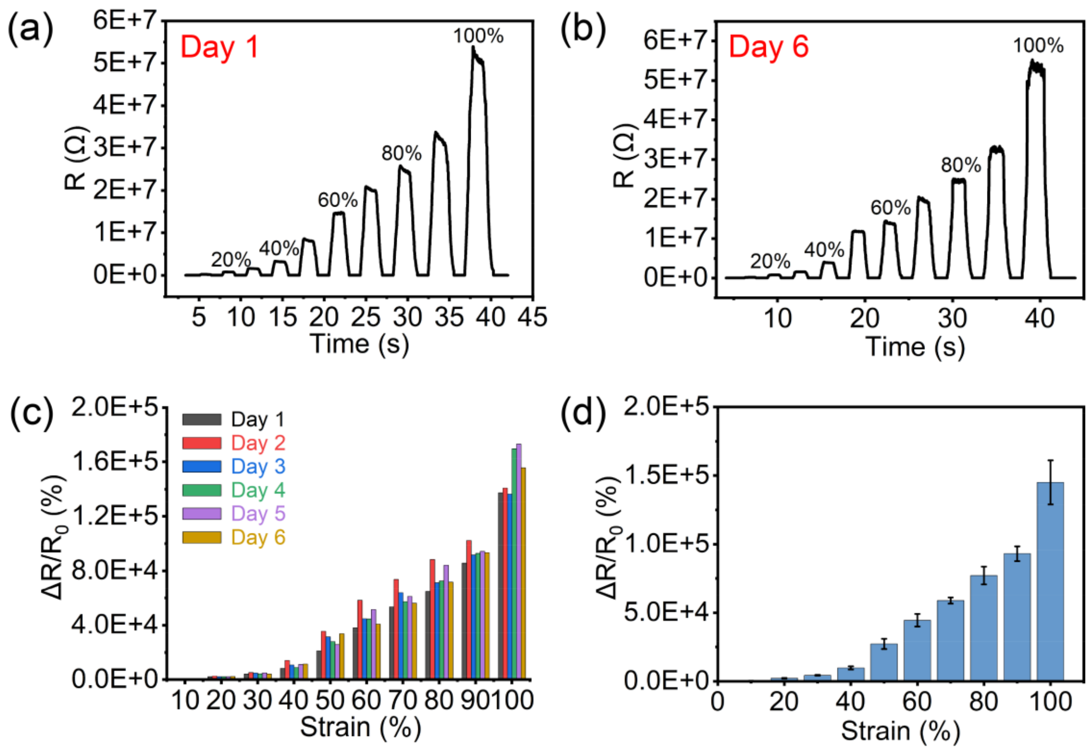

Figure S9. Dynamic resistance variation curve of the sensor to different strains (1-100%) in (a) Day 1 and (b) Day 6. (c) Resistance response versus strain bar graphs of the sensor for six days. (d) The average resistance response of the sensor to various strains within six days.

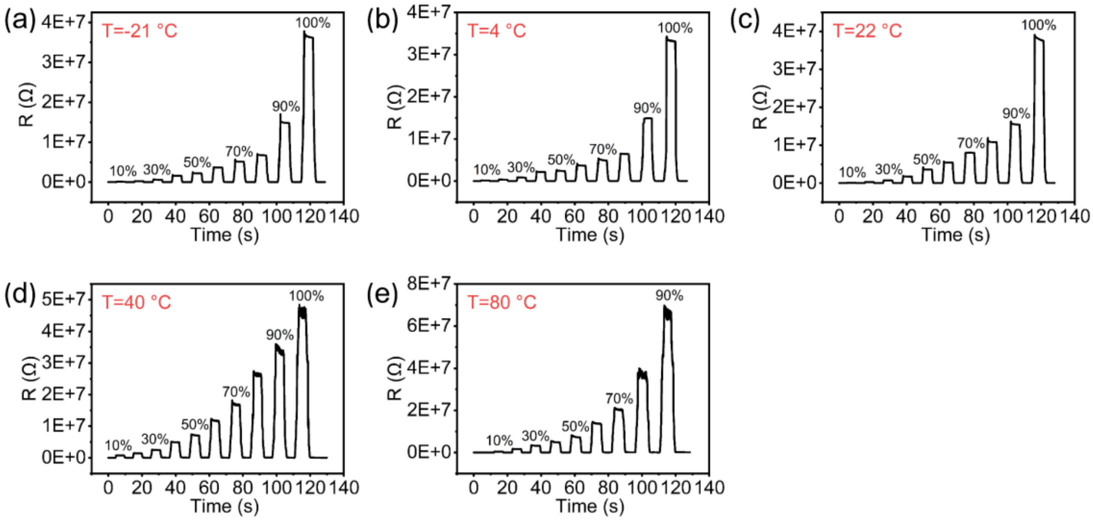

Figure S10. Time-dependent resistance variation of the sensor to 10%-100% strain after being placed at (a) -21, (b) 4, (c) 22, (d) 40 and (e) 80 °C for 1 h before testing.

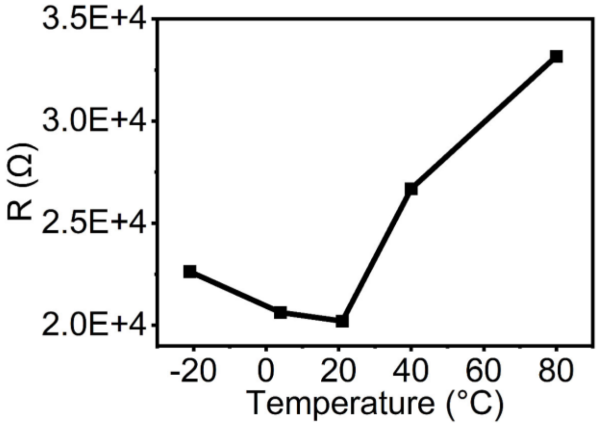

Figure S11. Plot of the initial resistance of the strain sensor versus temperature.

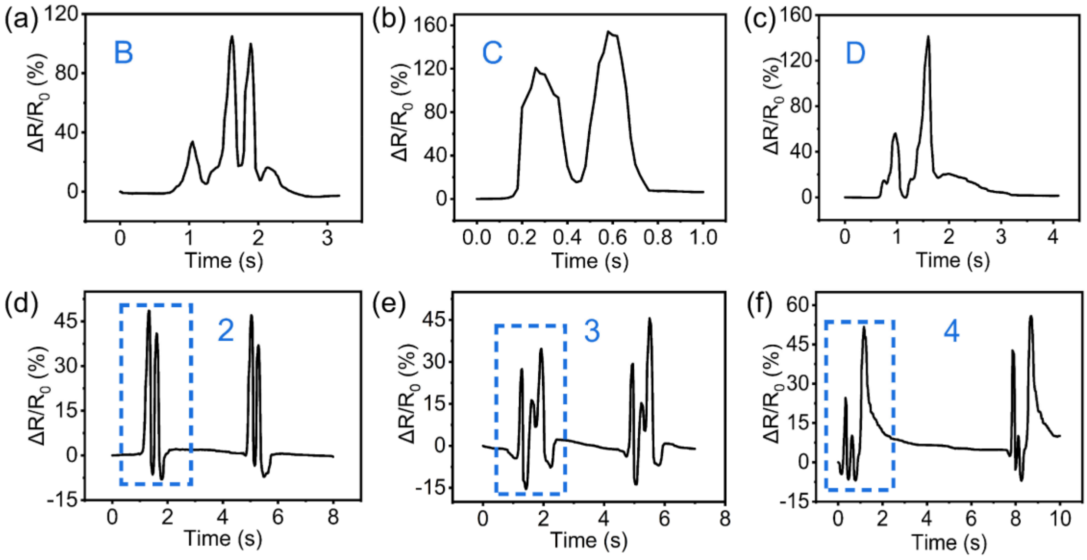

Figure S12. Dynamic responses of the sensor attached to the hand when writing different symbols: (a) “B”, (b) “C”, (c) “D”, (d) “2”, (e) “3”and (f) “4”. It is clear that the response patterns vary significant with different symbols.

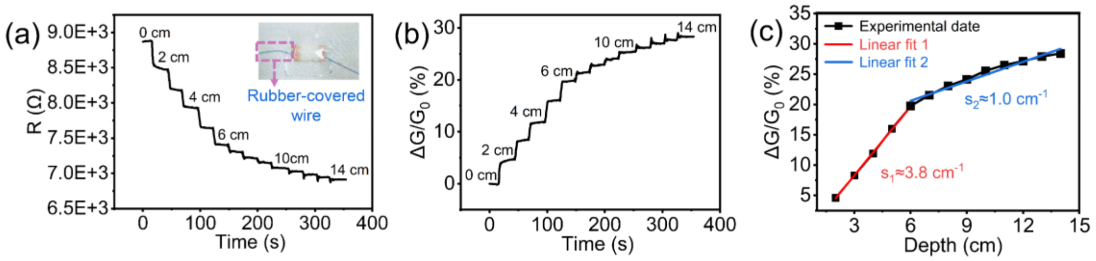

Figure S13. Measurement of the depth of water using the organohydrogel microsphere sensor with rubber-covered wires. (a) Dynamic resistance change of the sensor in response to different depth of water. (b) Dynamic conductance response of the sensor to different depth of water. (c) Corresponding response versus water depth curves, from which the sensitivity was derived by performing linear fitting.

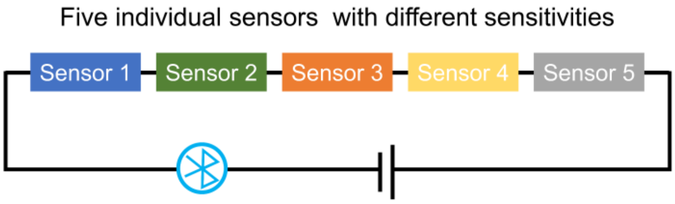

Figure S14. The circuit diagram of the wireless single-channel gesture recognition system used for recognizing the bending of different fingers by connecting five individual sensors with different sensitivities in series and attaching them to five fingers.

Table S1. The performance index of various hydrogel-based strain sensors corresponding to the radar diagram in **Fig. 4h**.

Sensing Materials	Sensitivity (Maximum gauge factor)	low detection limit	Operating temperature (°C)	Ref.
P(NIPAM-co-AA)	7.57	0.25%	RT	[1]
PAAN	17.9	0.05%	RT	[2]
SA/TA/PAM	9	0.05%	RT	[3]
SA/PAM	165	10%	RT	[4]
MXene/PAM/PVA	44.85	1%	-40-20	[5]
Ca-PAA-SA-CNTs	6.29	1%	RT -100	[6]
MXene/PAM	10.22	1%	RT	[7]
PANI-NPs/P(PEG-co-AA)	2.43	0.5%	RT	[8]
PAM/DEAP/PEGDA-200	6.44	2%	RT	[9]
PAM/MMT/CNTs	8.5	1%	-60-60	[10]
PAM organohydrogel microsphere	1275	0.05%	-21-80	This work

NIPAM, N-isopropyl acrylamide; AA, acrylic acid; PAAN, polyaniline precursor pre-infiltrating into poly (acrylic acid) hydrogel matrix; SA, sodium alginate; TA, tannic acid; PAM, polyacrylamide; PVA, polyvinyl alcohol; PAA, polyacrylic acid; CNTs, carbon nanotubes; PANI-NPs, polyaniline nanoparticles; PEG, poly (ethylene glycol) methacrylate; DEAP, 2,2-diethoxy acetophenone; PEGDA-200, polyethylene glycol 200 diacrylate; MMT, montmorillonite; RT, room temperature.

References

[1] Q. Liang, X. Xia, X. Sun, D. Yu, X. Huang, G. Han, S. M. Mugo, W. Chen, Q. Zhang, *Adv. Sci.* **2022**, *9*, 2201059.

[2] G. Su, S. Yin, Y. Guo, F. Zhao, Q. Guo, X. Zhang, T. Zhou, G. Yu, *Mater. Horiz.* **2021**, *8*, 1795-1804.

[3] H. Qiao, P. Qi, X. Zhang, L. Wang, Y. Tan, Z. Luan, Y. Xia, Y. Li, K. Sui, *ACS Appl. Mater. Interfaces* **2019**, *11*, 7755-7763.

[4] H. Xu, Y. Lv, D. Qiu, Y. Zhou, H. Zeng, Y. Chu, *Nanoscale* **2019**, *11*, 1570-1578.

[5] H. Liao, X. Guo, P. Wan, G. Yu, *Adv. Funct. Mater.* **2019**, *29*, 1904507.

[6] J. Wei, J. Xie, P. Zhang, Z. Zou, H. Ping, W. Wang, H. Xie, J. Z. Shen, L. Lei, Z. Fu, *ACS Appl. Mater. Interfaces* **2021**, *13*, 2952-2960.

[7] L. Guan, H. Liu, X. Ren, T. Wang, W. Zhu, Y. Zhao, Y. Feng, C. Shen, A. V. Zvyagin, L. Fang, B. Yang, Q. Lin, *Adv. Funct. Mater.* **2022**, *32*, 2112281.

[8] X. Yu, H. Zhang, Y. Wang, X. Fan, Z. Li, X. Zhang, T. Liu, *Adv. Funct. Mater.* **2022**, 2204366.

[9] C. Hang, X. Zhao, S. Xi, Y. Shang, K. Yuan, F. Yang, Q. Wang, J. Wang, D. W. Zhang, H. Lu, *Nano Energy* **2020**, *76*, 105064.

[10] H. Sun, Y. Zhao, S. Jiao, C. Wang, Y. Jia, K. Dai, G. Zheng, C. Liu, P. Wan, C. Shen, *Adv. Funct. Mater.* **2021**, *31*, 2101696.
